# Pharmacologic Inhibition of COX-1 and COX-2 in Influenza A Viral Infection in Mice

**DOI:** 10.1371/journal.pone.0011610

**Published:** 2010-07-15

**Authors:** Michelle A. Carey, J. Alyce Bradbury, Yvette D. Rebolloso, Joan P. Graves, Darryl C. Zeldin, Dori R. Germolec

**Affiliations:** Division of Intramural Research, National Institute of Environmental Health Sciences, National Institutes of Health, Research Triangle Park, North Carolina, United States of America; Karolinska Institutet, Sweden

## Abstract

**Background:**

We previously demonstrated that cyclooxygenase (COX)-1 deficiency results in greater morbidity and inflammation, whereas COX-2 deficiency leads to reduced morbidity, inflammation and mortality in influenza infected mice.

**Methodology/Principal Findings:**

We investigated the effects of COX-1 and COX-2 inhibitors in influenza A viral infection. Mice were given a COX-1 inhibitor (SC-560), a COX-2 inhibitor (celecoxib) or no inhibitor beginning 2 weeks prior to influenza A viral infection (200 PFU) and throughout the course of the experiment. Body weight and temperature were measured daily as indicators of morbidity. Animals were sacrificed on days 1 and 4 post-infection and bronchoalveolar lavage (BAL) fluid was collected or daily mortality was recorded up to 2 weeks post-infection. Treatment with SC-560 significantly increased mortality and was associated with profound hypothermia and greater weight loss compared to celecoxib or control groups. On day 4 of infection, BAL fluid cells were modestly elevated in celecoxib treated mice compared to SC-560 or control groups. Viral titres were similar between treatment groups. Levels of TNF-α and G-CSF were significantly attenuated in the SC-560 and celecoxib groups versus control and IL-6 levels were significantly lower in BAL fluid of celecoxib treated mice versus control and versus the SC-560 group. The chemokine KC was significantly lower in SC-560 group versus control.

**Conclusions/Significance:**

Treatment with a COX-1 inhibitor during influenza A viral infection is detrimental to the host whereas inhibition of COX-2 does not significantly modulate disease severity. COX-1 plays a critical role in controlling the thermoregulatory response to influenza A viral infection in mice.

## Introduction

Seasonal influenza is a major cause of morbidity and mortality worldwide. The outbreak of highly pathogenic H5N1 avian influenza in 2003 and the recent emergence of a new triple-reassortant influenza A (H1) virus — containing genes from avian, human, and swine influenza viruses [1] —are important reminders of the public health and clinical challenges posed by influenza viruses. Because of antigenic drift and shift of influenza viruses, new epidemics are difficult to prevent or control and vaccines need to be updated annually [2], [3]. There is a clear need for effective alternative or complementary therapies to vaccines and antiviral agents. A better understanding of the endogenous regulatory pathways modulating host response may pave the way for better therapies in the future.

Previous studies have suggested that dysregulation of the host inflammatory response to influenza may contribute to disease severity [4], [5]. Many severe infections are characterized by excessive inflammation and elevated pro-inflammatory cytokines and chemokines, a phenomenon known as hypercytokinemia or “cytokine storm.” Such findings have prompted suggestions that immunomodulatory and anti-inflammatory agents might be effective for treatment and prophylaxis of influenza [6]–[8].

The cyclooxygenase (COX) enzymes, which catalyze the conversion of arachidonic acid into prostaglandins, play a significant role in modulating inflammation and immune responses [9]–[14]. COX inhibitors are used clinically for their anti-inflammatory, analgesic and anti-pyretic properties and include the conventional non-selective non-steroidal anti-inflammatory drugs (NSAIDs) (e.g. ibuprofen) and COX-2 selective inhibitors (e.g. celecoxib).

We previously found that COX-1 deficiency is detrimental whereas COX-2 deficiency is beneficial to the host in response to influenza A virus; infection induced less severe illness in COX-2^–/–^ mice compared to WT and COX-1^–/–^ mice, and inflammation was elevated in the COX-1^–/–^ mice but ameliorated in the COX-2^–/–^ mice [11]. These findings suggest important but somewhat contrasting roles for both COX isoforms in the host response to influenza A virus. The effects of selective pharmacologic inhibitiors of COX-1 or COX 2 in this model have not been investigated. Hence, the objective of the present study was to examine the effects of pharmacologic inhibition of COX-1 or COX-2 on the host response to acute influenza A viral infection in mice.

## Results

### Clinical signs of infection

**Figure 1:**
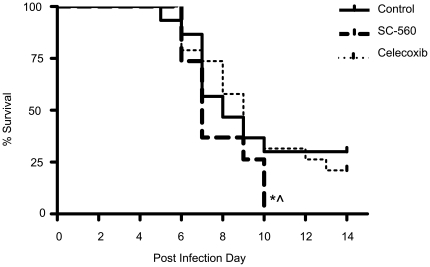
Mortality following influenza A viral infection in control (n = 30), SC-560 (n = 19) and celecoxib (n = 19) treatment groups; *p<0.05 versus control, ∧ p<0.05 versus celecoxib.

By day 10 of infection, 100% of the SC-560 group had succumbed to the illness. In contrast, on day 10, there were significantly more survivors in the no inhibitor and celecoxib treated groups where only 70% and 68%, respectively, succumbed to infection (p<0.05) (Figure 1).

**Figure 2:**
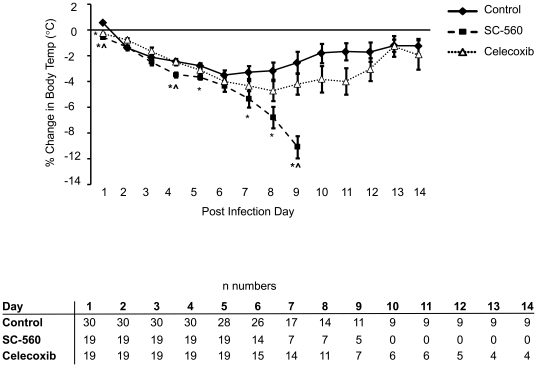
Time course of body temperature changes following influenza A viral infection in control, SC-560 and celecoxib treatment groups. Numbers of mice in each group and timepoint are shown in the table. Data represent mean ± SEM; *p<0.05 versus control.

Body temperature increased slightly in the control group on day 1 of infection (Figure 2). From days 2 to 6, mice in this group became progressively hypothermic. After day 6, body temperature stabilized in surviving animals and trended upwards toward pre-infection levels through day 14 post-infection. The temperature profile of celecoxib treated mice was similar to that of control mice except that on day 1, body temperature had already dropped in the celecoxib treated group and was significantly lower than the control group. Body temperature also dropped in the SC-560 treated mice on day 1 of infection; however, compared to control and celecoxib treated groups, body temperature dropped at a more rapid rate in the SC-560 group from days 1 to 9 post-infection (Figure 2).

**Figure 3:**
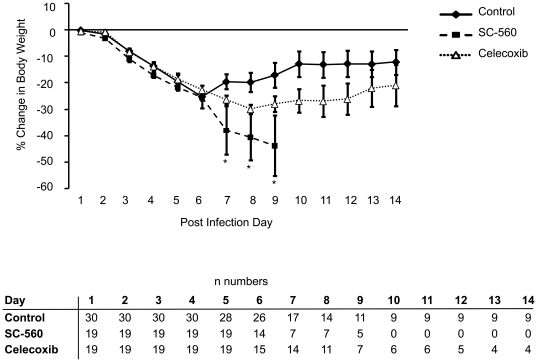
Time course of body weight changes following influenza A viral infection in control, SC-560 and celecoxib treatment groups. Numbers of mice in each group and timepoint are shown in the table. Data represent mean ± SEM; *p<0.05 versus control.

The control group began to lose weight on day 1 post-infection and progressively lost more weight until day 7 when surviving animals in this group began to regain weight (Figure 3). A similar pattern of weight change was observed in the celecoxib treated group; although there tended to be greater weight loss in the celecoxib group versus the control group, the differences were not statistically significant. SC-560 treated mice lost weight at a similar rate to the control and celecoxib groups until day 7 post-infection when the SC-560 group began to rapidly lose weight until day 10 when there were no surviving animals left in this group (Figure 3).

Taken together, these data demonstrate that COX-1 inhibition, but not COX-2 inhibition, results in increased mortality, more severe hypothermia and increased weight loss following influenza A viral infection.

### BAL fluid inflammatory cells

**Figure 4:**
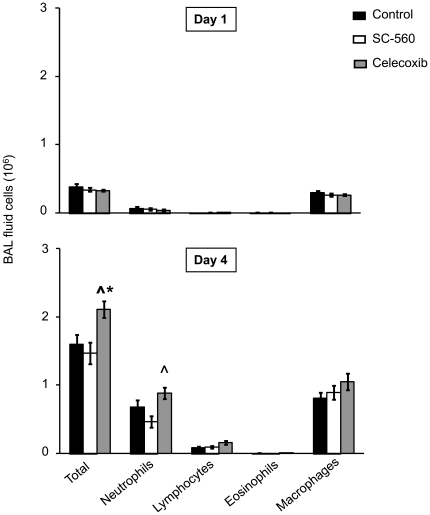
BAL fluid cellularity on days 1 and 4 of infection in control, SC-560 and celecoxib treatment groups. Data represent mean ± SEM (n = 16–17 per group and timepoint). *p<0.05 versus control, ∧ p<0.05 versus SC-560.

In all three groups on day 1 post-infection, BAL fluid contained mainly macrophages with a small number of infiltrating neutrophils (Figure 4, Table 1). On day 4 post-infection, BAL fluid cell influx increased in all three groups; however, there was a modest but statistically significant elevation in absolute numbers of total cells and neutrophils in BAL fluid of celecoxib treated mice versus control and SC-560 treated mice (p<0.05). There were no significant differences between the groups in percentages of different cell type at either timepoint (Table 1).

**Table 1:** Percentage cellular compostion of BAL fluid.

		Neutrophils	Lymphocytes	Eosinophils	Macrophages
**Day 1**	**Control**	15±5	2±0	1±1	82±5
	**SC-560**	15±4	3±1	2±1	80±4
	**Celecoxib**	13±3	3±1	2±1	82±4
**Day 4**	**Control**	40±5	6±1	0±0	54±5
	**SC-560**	32±5	6±1	0±0	62±5
	**Celecoxib**	43±5	7±1	1±0	49±4

N = 16–17 per group per timepoint; data shown are mean ± SEM; no significant differences between treatment groups for each timepoint.

### Lung Viral Titres

**Figure 5:**
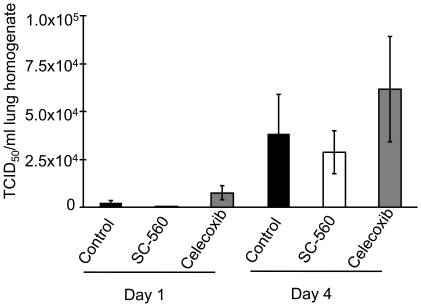
Lung viral titres on days 1 and 4 of infection. Data represent mean ± SEM (n = 8–10 per group and timepoint). Differences between the three treatment groups are not statistically significant; however, viral titres on day 4 are significantly higher than on day 1 for each group.

Virus was detectable in the lungs of all three groups on day 1 of infection (Figure 5). By day 4, viral titres had markedly increased. There were no significant differences between the three groups on either day.

### BAL fluid and serum cytokines

On day 1 post-infection, all cytokines tested, with the exception of IL-1β, were detectable and there were no significant differences between the three groups (Table 2). By day 4, there was a marked elevation in BAL fluid cytokine levels in all three groups. There were no significant differences between the groups in the BAL fluid levels of MCP-1, IL-1β, IFN-γ, IL-12p40 or MIP-1α. BAL fluid levels of TNF-α and G-CSF were significantly attenuated in the SC-560 and celecoxib treated groups versus control. The chemokine KC was also significantly lower in the SC-560 treated group versus control. In addition, BAL fluid levels of IL-6 were significantly lower in celecoxib treated mice versus control. Similar to BAL fluid, levels of G-CSF in serum on day 4 were significantly lower in the celecoxib treated group versus control; there was a trend for reduced levels in the SC-560 treated group versus control on day 5. There were no significant differences between the groups in serum levels of other cytokines (Table 2). Taken together, these data demonstrate that the increased morbidity and mortality in COX-1 inhibitor treated mice is accompanied by reduced BAL fluid levels of pro-inflammatory cytokines and chemokines. Inhibitor treatment had less of an effect on circulating cytokine levels compared to BAL fluid cytokine levels.

**Table 2:** BAL fluid and serum cytokine/chemokine levels (pg/ml).

Test material	Analyte	Day 1			Day 4		
		Control	SC-560	Celecoxib	Control	SC-560	Celecoxib
**BAL fluid**	**MCP-1**	14±10	12±4	2±1	691±85	638±94	679±89
	**IL-1β**	0±0	0±0	0±0	6±1	3±1	5±1
	**IFN-γ**	1±0	1±0	1±0	5±1	4±1	4±1
	**TNF-α**	4±1	4±2	4±1	49±10	29±4*	33±5*
	**IL-6**	6±4	2±0	1±0	271±43	248±58	145±18* ∧
	**IL-12p40**	6±1	9±2	15±6	591±123	529±101	538±98
	**G-CSF**	5±2	4±1	4±1	387±55	203±31*	253±46*
	**KC**	7±2	11±2	12±3	101±13	70±11*	87±11
	**MIP-1α**	25±9	26±6	16±5	91±15	74±15	89±13
**Serum**	**MCP-1**	304±41	315±22	318±31	307±15	306±28	353±43
	**IL-1β**	16±3	22±8	26±7	25±6	24±2	15±4
	**IFN-γ**	166±23	200±34	206±35	153±22	149±32	228±57
	**TNF-α**	636±102	800±191	814±99	769±149	522±94	751±160
	**IL-6**	43±7	40±4	39±4	58±8	43±4	45±5
	**IL-12p40**	471±48	516±92	597±165	652±94	446±73	618±68
	**G-CSF**	103±12	101±9	76±9	187±19	139±16	126±16*
	**KC**	70±14	56±9	52±11	174±41	123±17	93±12
	**MIP-1α**	413±74	525±153	500±95	498±106	580±72	437±95

n = 10–17 per group per timepoint; data shown are mean ± SEM.

* p<0.05 vs. Control Day 4.

∧ p<0.05 vs. SC-560 Day 4.

## Discussion

The present study examined the effects of treatment with a COX-1 or COX-2 selective inhibitor on the host response to influenza A viral infection in mice. Treatment with the COX-1 selective inhibitor SC-560 was associated with greater mortality and greater infection-induced changes in body temperature and body weight compared to treatment with the COX-2 selective inhibitor celecoxib or no inhibitor (control). Numbers of inflammatory cells in the BAL fluid were increased in the celecoxib treated group compared to the SC-560 and control groups. Inhibition of either COX enzyme led to decreases in BAL fluid levels of TNF-α and G-CSF. In contrast only inhibition of COX-1 led to a decrease in BAL fluid levels of KC and only inhibition of COX-2 led to a decrease in levels of IL-6. Viral titres were similar between the treatment groups.

We previously demonstrated a biphasic temperature response to influenza viral infection in mice — an initial hyperthermic response followed by a progressive hypothermic response [11]. Mice and other small rodents tend to develop hypothermia rather than fever in response to infectious stimuli [15]. In the present study, inhibition of either COX-1 or COX-2 blocked the initial hyperthermic response. Subsequently, inhibition of COX-1 led to profound hypothermia whereas inhibition of COX-2 led to a normal hypothermic response. These results contrast with observations in COX-deficient mice in that COX-1 deficiency led to a greater hyperthermic response and COX-2 deficiency abrogated the development of hypothermia [11]. The present findings are similar to the prior study with knockout mice in that COX-2 deficiency abolished the hyperthemic response and COX-1 deficiency worsened the degree of hypothermia. Considerable evidence supports the role of COX enzymes in thermoregulation. COX-2 has predominantly been implicated in modulating body temperature changes in response to infection. However, some studies have also implicated COX-1. Studies in rats have shown that LPS-induced hypothermia is blocked by the COX-1 inhibitors SC-560 [16], [17] and valeryl salicylate [16], but enhanced by the COX-2 inhibitor SC-236 [17]. In contrast, an earlier study, also in rats, found that the COX-2 inhibitor SC-236 blocked LPS-induced hyperthermia but that the COX-1 inhibitor SC-560 resulted in profound hypothermia in response to LPS [18]. The results of the latter study are consistent with the observations of the current study.

A critical challenge for the immune system is balancing the immune response to control infection while minimizing damage to the host. It is thought that much of the morbidity and mortality associated with influenza infection can be attributed to an over exuberant immune response leading to excessive production of cytokines and excessive inflammation at the site of infection [5], [19]–[21]. Indeed, when we examined the response to influenza A virus in the COX deficient mice, clinical signs of infection correlated with the inflammatory response [11]. Inflammation was reduced in COX-2 deficient mice and this was consistent with less morbidity. In contrast, inflammation in COX-1 deficient mice was enhanced and this was associated with a poorer clinical outcome. Surprisingly, in the present study clinical signs of infection in the COX inhibitor treated mice did not correlate with inflammation. The celecoxib treated group had mildly elevated levels of inflammatory cells in the BAL fluid and several pro-inflammatory cytokines remained unchanged whereas others were reduced compared to the control group. There was little difference between the celecoxib treated and control groups with respect to clinical signs. In contrast, SC-560 treatment had no significant effect of BAL fluid inflammatory cell numbers, although there was a trend for decreased neutrophils which may be related to the depressed levels of the neutrophil chemokine KC on day 4 of infection. Otherwise, SC-560 treatment produced a very similar BAL fluid cytokine profile to celecoxib treatment. Yet, the SC-560 group exhibited more severe clinical signs of illness and 100% mortality. These results suggest that inflammation can be dissociated from clinical outcome in the COX inhibitor treated animals.

In addition to immune system activation, inflammatory or infectious stimuli induce a highly coordinated central nervous system response which modulates body temperature changes. The COX-1 inhibitor induced profound hypothermia in influenza infected mice suggesting that COX-1 is required for the suppression of hypothermia following infection with influenza in mice. The degree of hypothermia in mice can predict mortality: when body temperature drops below a certain point in various infection models, death is almost inevitable [22], [23]. It is difficult to say definitively whether the hypothermia was the cause of excessive mortality in the SC-560 or a consequence/marker of some other process.

The mechanisms regulating hypothermia are not fully understood but cytokines such as TNF-α and interleukins have been shown to induce or modulate the hypothermic response [24]. Studies have shown that the degree of hypothermia correlates with levels of certain pro-inflammatory cytokines [25]. We did not observe any elevation in cytokine levels in the SC-560 treated group relative to control that might explain the excessive hypothermia; cytokines examined were either unchanged or blunted in relation to control.

The differential effects of SC-560 on the temperature response may in fact be mediated further upstream in the process (i.e. centrally in the brain). Several studies support a role of COX products in the central nervous system response to infectious stimuli. For example, microinjection of a COX inhibitor into the preoptic area of rat brain, a region believed to be responsible for thermoregulation, decreased the fever response to LPS suggesting that prostaglandin biosynthesis in that region of the brain is necessary to modulate the thermoregulatory response to infection [26]. PGE_2_ produces fever when injected intracerebroventricularly [27] and mice lacking the EP3 receptor also lacked an appropriate febrile response to PGE2 [28]. Oka and colleagues found that the EP3 receptor is necessary to produce fever and also necessary to prevent profound hypothermia in response to LPS [29]. The effects of COX inhibitors on the brain circuitry that is activated as part of the thermoregulatory response to infection in mice have not been thoroughly investigated and would be an interesting area for future study.

Treatment of influenza A virus infected mice with selective COX inhibitors did not recapitulate the phenotypes observed using the same model in COX knockout mice. This is not a unique observation: we have previously observed discordance between studies with COX knockout mice and COX inhibitor treated mice in an allergic airway disease model [10], [30]. There are several potential explanations for the discordance. First, in inhibitor studies, the COX enzymes are inhibited at the time of study, whereas COX knockout mice are genetically deficient in the enzyme from the point of conception. Second, the COX knockout mice have a total absence of the respective COX activity, a condition likely not attainable, even with higher doses of COX inhibitors [31]. Third, the COX enzymes are known to play differential roles in immune development [12], [13] and thus immune phenotypes of the COX knockout mice may be a consequence of developmental effects of COX deficiency rather than inhibition of the enzyme. Finally, it is also possible that some of the discordance between the knockout and inhibitor studies could be due to COX-independent effects of the inhibitors [32], [33].

An interesting observation was that treatment with either COX inhibitor led to depressed levels of G-CSF in BAL fluid on day 4 of infection and a similar trend was observed in serum. G-CSF expression is often induced during infection and is thought to play an important role in the regulation of the systemic and local neutrophil response to the infection [34], a process known as stress or emergency granulopoiesis. However, the depressed levels of G-CSF in the COX inhibitor treated groups appear not to have significantly affected BAL fluid neutrophil levels; in fact, neutrophils were slightly elevated on day 4 in the celecoxib treated group. It is possible that the importance of G-CSF in regulating stress granulopoiesis is pathogen/route dependent: in G-CSF null mice, neutrophilia is normal in response to intravenous *Candida albicans* or *intraperitoneal Listeria monocytogenes* but blunted in response to intravenous *Listeria monocytogenes* [35]–[37]. Nevertheless, the observation that COX inhibitors can depress G-CSF levels during stress granulopoiesis is important and may have significant consequences in other infectious models/states.

The COX enzymes are a major pharmaceutical target. Because of their analgesic effects and their potent anti-inflammatory and anti-pyretic properties, NSAIDs are amongst the most widely prescribed drugs in the western world. The classical NSAIDs inhibit both COX-1 and COX-2 but tend to be more selective towards COX-1 [38]. While one cannot extrapolate from animal studies directly to human populations, the profound effect of the COX-1 inhibitor SC-560 on the thermoregulatory response to influenza A virus is noteworthy and deserves more detailed study. A key focus of future studies will be to examine how the thermoregulatory response in the brain of influenza A virus infected mice is modulated by SC-560 and other COX inhibitors.

In the present study the COX inhibitors were administered orally and in chow given *ad libitum*. As we observed, the model was characterized by weight loss and so it is possible that mice were not receiving a consistent dose of drug each day. Nevertheless, the fact that the COX-1 inhibitor had such a profound effect with the greatest weight loss suggests the COX-1 is critical to host response to influenza viral infection and can still have a potent, long lasting effect, even at lower doses.

In summary, we have shown that treatment with a COX-1 inhibitor during influenza A viral infection is detrimental to the host, whereas treatment with a COX-2 selective inhibitor does not significantly modulate disease severity. Furthermore, our studies point to a critical role for COX-1 in controlling the thermoregulatory response to influenza infection in mice.

## Materials and Methods

### Animals and drug treatments

All animal studies were conducted in accordance with principles and procedures outlined in the National Institutes of Health Guide for the Care and Use of Laboratory Animals and were approved by the Animal Care and Use Committee at the National Institute of Environmental Health Sciences (NIEHS) (Protocol 06-08 LRB, assurance number A4149-1). Female, pathogen-free, 3–5 mo old mice were of a hybrid C57BL/6J×129/Ola genetic background bred at Taconic Farms. They were housed under identical conditions and fed NIH 31 rodent chow (Agway) *ad libitum*. NIH-31 rodent chow was formulated into meal containing either 1500 ppm of the COX-2 selective inhibitor celecoxib (LKT laboratories, St. Paul, MN), 20 ppm of the COX-1 selective inhibitor SC-560 (Cayman Chemical, Ann Arbor, MI) or no inhibitor at Research Triangle Institute (Research Triangle Park, NC). Mice were fed control or COX-inhibitor containing diet *ad libitum* beginning 2 weeks prior to infection and continuing through the duration of each experiment. Previous studies have demonstrated that similar doses of COX inhibitors are well tolerated and result in selective inhibition of the respective COX isoforms in mice [39].

### Influenza infection model (Figure 6)

**Figure 6:**
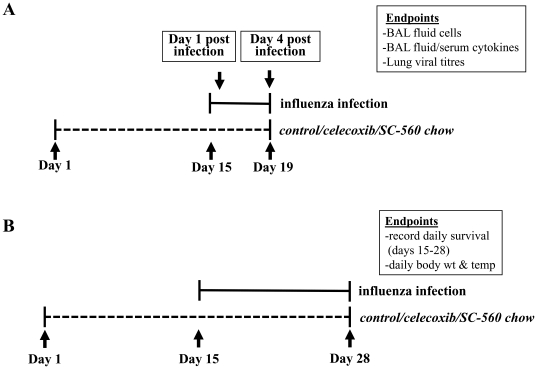
Overview of study design. (A) Inflammatory endpoints. (B) Clinical endpoints.

Two weeks following the initiation of COX inhibitor treatment (day 0), mice were weighed and rectal temperatures were recorded electronically (Thermalert TH-5; Physitemp). A frozen aliquot of influenza A/Hong Kong/8/68 (H3N2) was used to prepare dilutions in HBSS containing 200 PFU in 50 µl. The virus was a generous gift from Dr. R. Luebke (U.S. Environmental Protection Agency, Research Triangle Park, NC). Mice were lightly anesthetized with isofluorane and infected by intranasal instillation of 25 µl/nostril. There were 2 experimental groups:

*Group 1*: BAL was performed on days 1 or 4 post-infection for the measurement of BAL fluid cells, cytokines and viral titres (Figure 6A)
*Group 2*: Daily body temperature, body weight and mortality were recorded up to 2 weeks post-infection (Figure 6B).

### Bronchoalveolar lavage (BAL) fluid and serum processing and analysis

Mice were anesthetized by i.p. injection of sodium pentobarbital (80 mg/kg). A blood sample was drawn from the abdominal aorta. Serum was extracted, frozen and stored at −80°C. Lungs were lavaged with two 1-ml aliquots of HBSS that were subsequently combined. Approximately 90% of the total instilled volume was consistently recovered. The BAL fluid was placed on ice and centrifuged at 360×*g* for 10 min at 4°C. Aliquots of BAL fluid for cytokine analyses were stored at −80°C. Cells were resuspended in 1 ml of HBSS and counted using a Coulter counter (Z1 model; Coulter Electronics). Slides of BAL fluid cells were prepared (Cytospin 3; Shandon), stained with Wright-Giemsa (Fisher Scientific), and differentiated using conventional morphological criteria in a blinded fashion. Cytokine levels in BAL fluid and serum were determined with a Bio-Plex mouse cytokine kit (Bio-Rad, Hercules, CA) using fluorescently-labeled microsphere beads and a Bio-Plex suspension array system (Bio-Rad) according to the manufacturer's instructions.

### Pulmonary virus quantitation

On days 1 and 4 of infection, lungs were homogenized in ice-cold HBSS (10% w/v). The homogenates were centrifuged at 1000×*g* for 30 min to remove cell debris and the supernatants were stored at −80°C until assay. The tissue culture ID_50_ (TCID_50_) of virus in the lungs was determined as previously described [11]. Briefly, confluent monolayers of Madin-Darby canine kidney cells on 96-well microtiter plates were infected with one-half log_10_ dilutions of lung homogenates. After 3–4 days of incubation at 37°C, the wells were observed for cytopathic effect. The wells with cytopathic effect were counted, and the TCID_50_ was calculated according to the Reed-Muench method [40].

### Statistical analyses

Results are expressed as means ± SEM. Groups were compared by ANOVA followed by multiple comparison of means with Newman-Keuls multiple comparison test. An unpaired t-test was used when groups of only two were being compared. Survival was analyzed using the χ^2^ test with Fisher's exact method. All statistics were performed using GraphPad Prism (version 4) statistical software (GraphPad Software). Values of p<0.05 were considered significant.
